# Common modular architecture across diverse cortical areas in early development

**DOI:** 10.1073/pnas.2313743121

**Published:** 2024-03-06

**Authors:** Nathaniel J. Powell, Bettina Hein, Deyue Kong, Jonas Elpelt, Haleigh N. Mulholland, Matthias Kaschube, Gordon B. Smith

**Affiliations:** ^a^Optical Imaging and Brain Sciences Medical Discovery Team, Department of Neuroscience, University of Minnesota, Minneapolis, MN 55455; ^b^Center for Theoretical Neuroscience, Zuckerman Institute, Columbia University, New York, NY 10027; ^c^Frankfurt Institute for Advanced Studies, Frankfurt am Main 60438, Germany; ^d^Department of Computer Science and Mathematics, Goethe University, Frankfurt am Main 60629, Germany; ^e^International Max Planck Research School for Neural Circuits, Frankfurt am Main 60438, Germany

**Keywords:** network, cortex, development

## Abstract

In order to deal with a complex environment, animals form a diverse range of neural representations that vary across cortical areas, ranging from largely unimodal sensory input to higher-order representations of goals, outcomes, and motivation. The developmental origin of this diversity is currently unclear, as representations could arise through processes that are already area-specific from the earliest developmental stages or alternatively, they could emerge from an initially common functional organization shared across areas. Here, we use spontaneous activity recorded with two-photon and widefield calcium imaging to reveal the functional organization across the early developing cortex in ferrets, a species with a well-characterized columnar organization and modular structure of spontaneous activity in the visual cortex. We find that in animals 7 to 14 d prior to eye-opening and ear canal opening, spontaneous activity in both sensory areas (auditory and somatosensory cortex, A1 and S1, respectively), and association areas (posterior parietal and prefrontal cortex, PPC and PFC, respectively) showed an organized and modular structure that is highly similar to the organization in V1. In all cortical areas, this modular activity was distributed across the cortical surface, forming functional networks that exhibit millimeter-scale correlations. Moreover, this modular structure was evident in highly coherent spontaneous activity at the cellular level, with strong correlations among local populations of neurons apparent in all cortical areas examined. Together, our results demonstrate a common distributed and modular organization across the cortex during early development, suggesting that diverse cortical representations develop initially according to similar design principles.

## Significance

How the diversity of functional organization across brain areas emerges during development is unclear. By imaging spontaneous activity in both sensory and higher-order cortices, we find that a distributed and modular functional architecture with long-range correlations is a common feature of the developing cortex. This suggests that instead of displaying area-specific specializations already from early development, cortical areas that ultimately reflect diverse representations develop from an initially similar structure. These modular functional networks exhibit strong quantitative similarity across areas, suggesting that similar organizing principles might operate throughout the early cortex. Our findings therefore suggest a common modular organization might serve as a generic cortical substrate upon which later area-specific influences generate the functional specificity found in the mature brain.

The information represented by neural activity varies greatly across different regions of the neocortex. Neurons in primary sensory areas encode specific features of the external environment, for example, visual orientation (1) or auditory frequency (2), whereas neural activity in higher-order association areas represents complex aspects of both internal and external state, such as motivation and goal-directed planning (3, 4). The functional specification of these diverse cortical areas and their varied neural representations is thought to begin at the earliest stages of nervous system development with coarse gradients of gene expression that establish the rough layout and identity of cortical areas, which are then refined in an activity-dependent manner (5, 6). This refinement is initially driven by structured patterns of endogenously generated spontaneous activity and, subsequently, by sensory experience (7). The sources of these early cortical spontaneous patterns are themselves area specific, involving, for instance, spontaneous activity in modality-specific inputs from the sensory periphery such as the retina, cochlea, or whisker pad (8). Moreover, the onset of sensory stimulation varies greatly across sensory cortical areas, with orderly peripheral input to the somatosensory cortex already occurring prior to birth (9), whereas normal visual experience is only achieved with eye opening, which in several species, including mice and ferrets, takes place several weeks later (10).

Thus, in order to build a diversity of representations, the early functional organization of these endogenously generated networks might be expected to already vary considerably across areas of the developing cortex, tailored toward the area-specific representations of the mature brain. However, currently we lack a clear understanding of the degree to which the organization of these early networks, and the processes leading to the development of mature representations, actually varies across the neocortex. This leaves open an intriguing alternative possibility—that network structure across diverse cortical areas is initially shaped by generic and cortex-wide mechanisms, giving rise to a functional organization common to all cortical areas before later undergoing diversification through experience-dependent processes.

The columnar architecture in the primary visual cortex (V1) of primates and carnivores (11), such as the ferret, provides a particularly well-suited point of reference for exploring where the developing cortex operates on this spectrum between diversity and uniformity. Here, nearby neurons share similar selectivity for stimulus features, such as orientation, which are organized into repetitive patterns in which patches or modules of co-tuned neurons several hundred microns in diameter are distributed across the cortical surface, giving rise to the well-known maps of stimulus features (12–17). Notably, this distributed modular representational architecture is also reflected in the organization of functional networks during spontaneous activity, with strong correlations between co-tuned modules extending across millimeters (18–20). This modular spontaneous activity is already apparent during early development, where correlated network activity more than a week prior to eye opening predicts features of the future columnar orientation preference map (20).

Such a modular organization has been hypothesized to be a fundamental unit of cortical computation (reviewed in ref. 21). Indeed, functional modules reflecting stimulus features have been reported in other sensory cortices, including the visual areas V2 (22), V4 (23), MT (24), and IT (25). Likewise, although the functional mapping of stimulus features across the cortex is arguably less clear than in visual areas, functional modules have also been reported in the auditory cortex (26–28) and somatosensory cortex (29, 30), building on the roughly linear topographic mappings of cochleotopy (2) and somatotopy (31). Similarly, the anatomical clustering of inputs carrying distinct streams of information has been observed in higher-order areas, including the prefrontal cortex (PFC) (32). However, throughout most of the neocortex, little is known about the functional organization of cortical networks at an early stage in development and whether such networks exhibit this modular structure.

Therefore, in order to determine whether the modular functional organization that is a hallmark of V1 and already apparent at an early age is also present elsewhere in the early developing neocortex, we investigated the patterns of ongoing spontaneous activity in multiple distinct cortical areas on both millimeter and cellular scales, examining both primary sensory (auditory—A1, somatosensory—S1, and visual—V1) and higher-order association cortices (posterior parietal cortex—PPC and prefrontal cortex—PFC) in the ferret. Critically, measuring spontaneous activity allows us to both compare network organization across multiple brain regions without relying on precisely designed stimulation paradigms for each area, which is a considerable challenge beyond primary sensory areas; and also to examine network structure at an early stage in development when sensory drive is still limited. Using this approach, we demonstrate a common modular organization showing quantitative similarity at both the cellular and columnar scale across both sensory and association areas in early development. Additionally, in all areas examined, spontaneous activity exhibited distributed and modular correlations extending across millimeters, demonstrating the presence of a common functional network structure. Our results therefore indicate that highly diverse cortical areas emerge from a common architecture of functional organization in early development.

## Results

### Spontaneous Activity Is Modular across Diverse Cortical Areas.

**Fig. 1.**
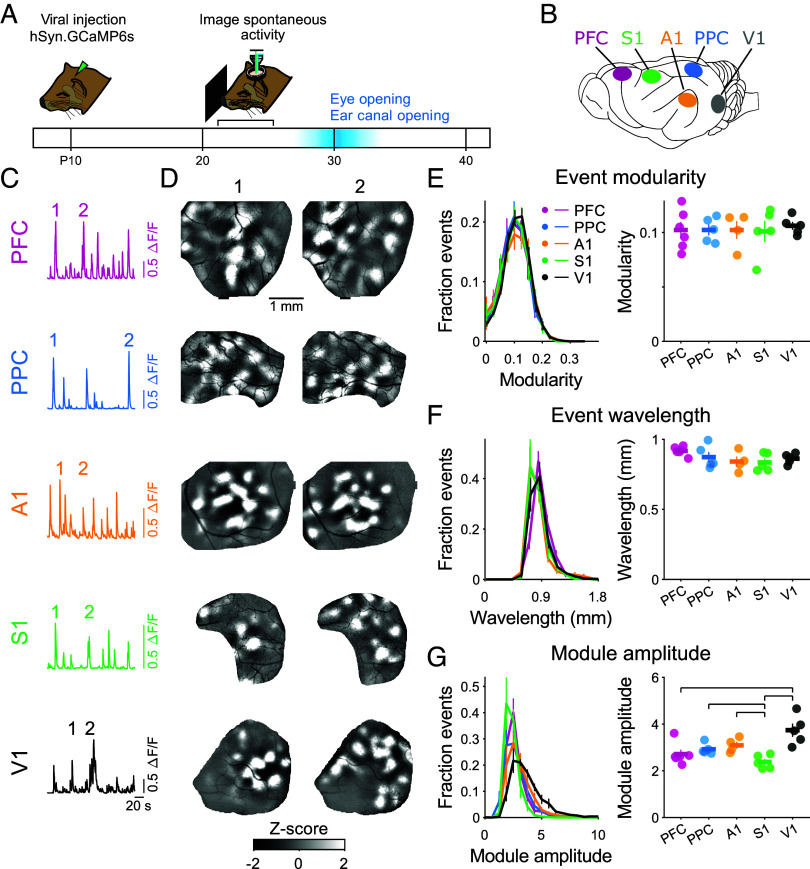
Spontaneous activity is highly modular in early development across diverse cortical areas. (*A*) Experimental schematic. Spontaneous activity was imaged at P21-24, 7 to 14 d prior to eye-opening and ear canal opening. (*B*) Activity was imaged in primary somatosensory (S1), auditory (A1), and visual (V1) cortices, and in the association areas PFC and PPC. (*C*) Time course of spontaneous activity (mean activity across ROI) in each brain area imaged in independent experiments. (*D*) Individual spontaneous events (times indicated in *C*) show highly modular activity in all areas. (*E*) The modularity of spontaneous events does not vary across cortical areas. For panels (*E*–*G*): *Left* plot shows distribution across all events, *Right* plot shows median of distribution for each animal (dots) and mean across animals (horizontal bar). (*F*) The wavelength of activity for spontaneous events is similar across events from different areas. (*G*) Module amplitude (active module vs. adjacent cortex) is generally similar across areas, with significantly lower amplitude in S1 and higher amplitude in V1. Significant post hoc pairwise comparisons indicated by horizontal lines. Error bars ± SEM.

To assess the mesoscopic functional organization in different cortical areas, we performed widefield imaging of virally expressed GCaMP6s (33) in the developing ferret cortex (P21-24), targeting both primary sensory (V1, A1, S1) and association areas (PPC, PFC) (Fig. 1 *A* and *B*). This age is approximately 7 to 14 d before eye-opening and ear canal opening in the ferret and is a time in which neural activity in V1 is both highly modular and already exhibits long-range correlations that reflect aspects of future columnar representations of visual features (20). Spontaneous activity was imaged under light isoflurane anesthesia, conditions which were previously shown in V1 to preserve the modular spatial characteristics of spontaneous activity found in awake animals (20).

We found that spontaneous activity in the primary sensory areas A1 and S1 (in addition to V1 as previously shown) exhibited pronounced modular spatial structure. Events consisted of multiple patches of elevated activity, each several hundred microns in diameter, that were distributed across the approximately 3-mm field of view (FOV) (Fig. 1 *C* and *D*, *SI Appendix*, Fig. S1, and Movies S1–S5). The patterns of active modules varied across spontaneous events in each area, showing a range of modular patterns. We next turned to the association areas PPC and PFC, where spontaneous activity in both regions was likewise strongly modular and appeared highly similar in structure to sensory areas (Fig. 1 *C* and *D*). In all areas, the temporal autocorrelations across activity decayed to near zero within 10 s (*SI Appendix*, Fig. S2), indicating the lack of temporal correlations on long timescales. Thus, spatially organized, modular patterns of functional activity appears to be a common feature shared across areas in the developing ferret cortex.

To quantify this modular structure, we first used the spatial autocorrelation function computed for each event to calculate event modularity across cortical areas, which assesses the regularity in size and spacing of patchy activity patterns (*Materials and Methods* and *SI Appendix*, Fig. S3 *A*–*C*). When examined across areas, we found that activity in all cortical regions exhibited highly significant modularity vs. surrogate controls drawn from frames without spontaneous events (25 of 25 FOVs significant vs. surrogate at *P* < 0.01, bootstrap test), that did not differ significantly across areas (Fig. 1*E*, (mean ± SEM) PFC: 0.10 ± 0.01 (n = 1,252 events from 6 animals, see *SI Appendix*, Table S1); PPC: 0.10 ± 0.01 (n = 1,228 events, 5 animals); A1: 0.10 ± 0.01 (n = 1,931 events, 4 animals); S1: 0.10 ± 0.01(n = 1,092 events, 5 animals); V1: 0.11 ± 0.01 (n = 1,971 events, 5 animals); Kruskal–Wallis (KW) test H(4) = 0.24, *P* = 0.993). We next computed the wavelength of modular activity from event autocorrelation patterns (*Materials and Methods* and *SI Appendix*, Fig. S3*C*), likewise finding that the spatial wavelength of events was also highly similar across cortical areas (Fig. 1*F*, (mean ± SEM) PFC: 0.92 ± 0.01 (n = 6 animals); PPC: 0.87 ± 0.04 (n = 5); A1: 0.84 ± 0.04 (n = 4); S1: 0.83 ± 0.03 (n = 5); V1: 0.86 ± 0.02 (n = 5); KW: H(4) = 7.33, *P* = 0.120). Notably, this common wavelength was similar to that observed for functional maps of orientation preference in mature V1 (34). To assess the degree to which activity was localized to modular patches vs. more diffuse and widespread activity, we computed the amplitude of activity within modular domains compared to surrounding cortex (termed “module amplitude”). Module amplitude was strong and generally similar across areas, although amplitude was significantly lower in S1 and higher in V1 relative to some other areas, indicating that, quantitatively, they exhibited subtle differences in their modular structure (Fig. 1*G*, (mean ± SEM) PFC: 2.72 ± 0.19 (n = 6 animals); PPC: 2.92 ± 0.10 (n = 5); A1: 3.09 ± 0.16 (n = 4); S1: 2.38 ± 0.13 (n = 5); V1: 3.74 ± 0.28 (n = 5); KW: H(4) = 16.28, *P* = 0.003; post hoc: PPC vs. S1: *P* = 0.0240, A1 vs. S1: *P* = 0.0101, V1 vs. S1: *P* = 0.0001, PFC vs. V1: *P* = 0.0011, *SI Appendix*, Table S3). Together, these results demonstrate that in the developing cortex, modular functional organization is a common feature that is shared across both sensory and association areas.

### Long-Range Correlated Networks Exist within Diverse Cortical Areas.

**Fig. 2.**
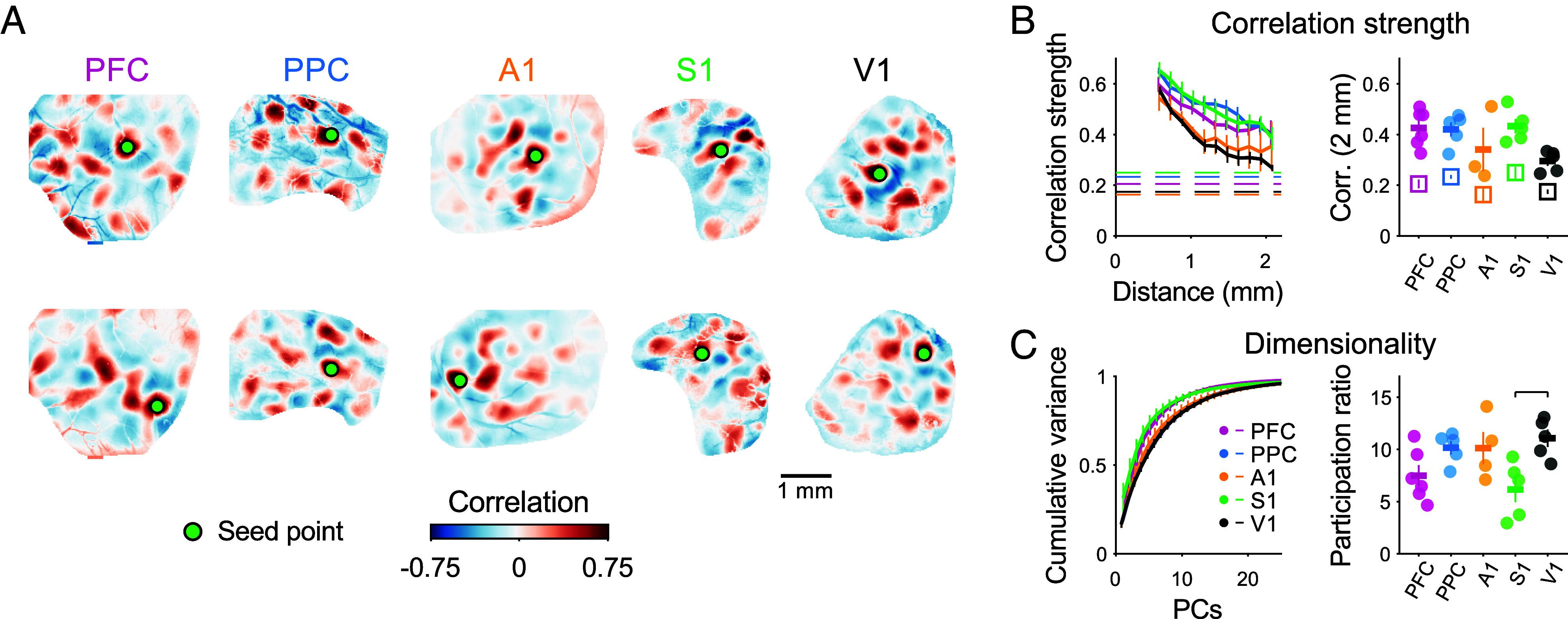
Diverse cortical areas show distributed and modular long-range correlations. (*A*) Correlations across spontaneous events reveal distributed and modular networks that extend across several millimeters in both sensory and association areas. Pixelwise correlations are shown for two different seed points (*Top/Bottom*), revealing the presence of multiple distributed modular networks within each cortical region. (*B*, *Left*) The strength of correlations declines with distance in all cortical areas, remaining statistically significant vs. surrogate controls (dashed lines, 1 per area) up to at least 2 mm, the limits of our imaging window. (*Right*) The strength of long-range correlations (1.8 to 2.2 mm away from seed point) is similar across areas. Dots show individual animals, horizontal bar indicates mean across animals. Open squares show mean ± SEM for surrogate controls. (*C*) Spontaneous activity is moderately low dimensional in all cortical areas. Variance explained by principal components (*Left*) and participation ratio of spontaneous activity (*Right*) are similar across areas. Significant post hoc pairwise comparisons indicated by horizontal lines. Error bars ± SEM.

A central feature of the functional organization in the visual cortex of carnivores and primates is that activity is not only modular, but it also exhibits long-range correlations in activity (35) that are distributed across the cortical surface such that specific sets of spatially distributed modules tend to be co-active. These long-range correlations define functional cortical networks and are already present in V1 in early development, going on to reflect aspects of the mapped selectivity for visual features (20) and corresponding clustered long-range horizontal connections present in mature animals, which are thought to link similar feature detectors across different locations in the visual field (36–38). It is possible that such a correlated network structure exists in other cortical areas that also exhibit modular activity. Alternatively, the activity of individual modules in these regions could be independent of each other, reflecting only local coherence within modular domains, but not the presence of millimeter-scale functional networks. To address this, we computed the spatial correlation of activity across all events imaged in each area. We found that in both sensory and association areas, the pattern of correlations over spontaneous events exhibited both strong positive and negative correlations that extended over multiple millimeters, covering our full imaging window (Fig. 2*A*). In all cases, the spatial pattern of correlations varied for different seed points within the FOV (Fig. 2*A* and *SI Appendix*, Fig. S4), indicating the presence of multiple distinct functionally correlated networks within each brain area.

We next compared the strength of these correlations as a function of distance, finding that correlations in spontaneous activity were statistically significant vs. surrogate data up to at least 2 mm away from the seed point (the limit imposed by our FOV size) in all areas examined (Fig. 2*B*; 24 of 24 FOVs significantly different from shuffle control, 1 A1 FOV excluded due to size limitations), and were similar across areas, with a non-significant trend toward weaker correlations in V1 relative to other areas (PFC: 0.43 ± 0.03 (mean ± SEM) (n = 6 animals); PPC: 0.42 ± 0.03 (n = 5); A1: 0.34 ± 0.09 (n = 3); S1: 0.43 ± 0.03 (n = 5); V1: 0.30 ± 0.02 (n = 5), KW:H(4) = 8.66 *P* = 0.070). Similar results were obtained when we alternatively assessed correlation strength through the variance of pixelwise correlations, and when controlling for the finite number of spontaneous events recorded in each imaging session (*SI Appendix*, Fig. S5).

The presence of these strong long-range correlations suggests that the patterns of active modules across spontaneous events occupy a constrained and low dimensional space of all possible activity patterns, where not all possible combinations of all possible module locations are equally likely to occur. To assess this, we computed the principal components (PCs) over all events within an area, finding that the leading PCs exhibited a clear modular structure (*SI Appendix*, Fig. S6), and that the majority of the variance across events could be explained by a relatively low number of PCs (Fig. 2 *C*, *Left*; PCs for 75% variance: [mean ± SEM) PFC: 6.0 ± 0.73 (n = 6 animals) (mean ± SEM); PPC: 8.8 ± 0.58 (n = 5); A1: 8.5 ± 0.96 (n = 4); S1: 6.0 ± 0.95 (n = 5); V1: 9.4 ± 0.68 (n = 5)]. We confirmed this by computing the participation ratio (39, 40), which provides a measure of the number of effective dimensions occupied by spontaneous activity patterns, thereby providing an estimate of the diversity of activity patterns across events. This measure roughly estimates the effective number of (linear) independent patterns present in spontaneous activity. Our analysis shows that spontaneous activity in all cortical areas examined resides in a moderately low dimensional space, with significantly lower dimensionality in S1 compared with V1 (Fig. 2 *C*, *Right*; (mean ± SEM) PFC: 7.48 ± 1.01 (n = 6 animals); PPC: 10.17 ± 0.66 (n = 5); A1: 10.13 ± 1.54 (n = 5); S1: 6.16 ± 1.21 (n = 5); V1: 11.07 ± 0.83 (n = 5); KW: H(4) = 10.85, *P* = 0.028; post hoc: V1 vs. S1 *P* = 0.029, *SI Appendix*, Table S4). Together, these results show that distributed networks with long-range modular correlations underlie a common functional organization that is highly similar across diverse cortical areas during early development.

### Modular Organization at Cellular Resolution throughout the Cortex.

**Fig. 3.**
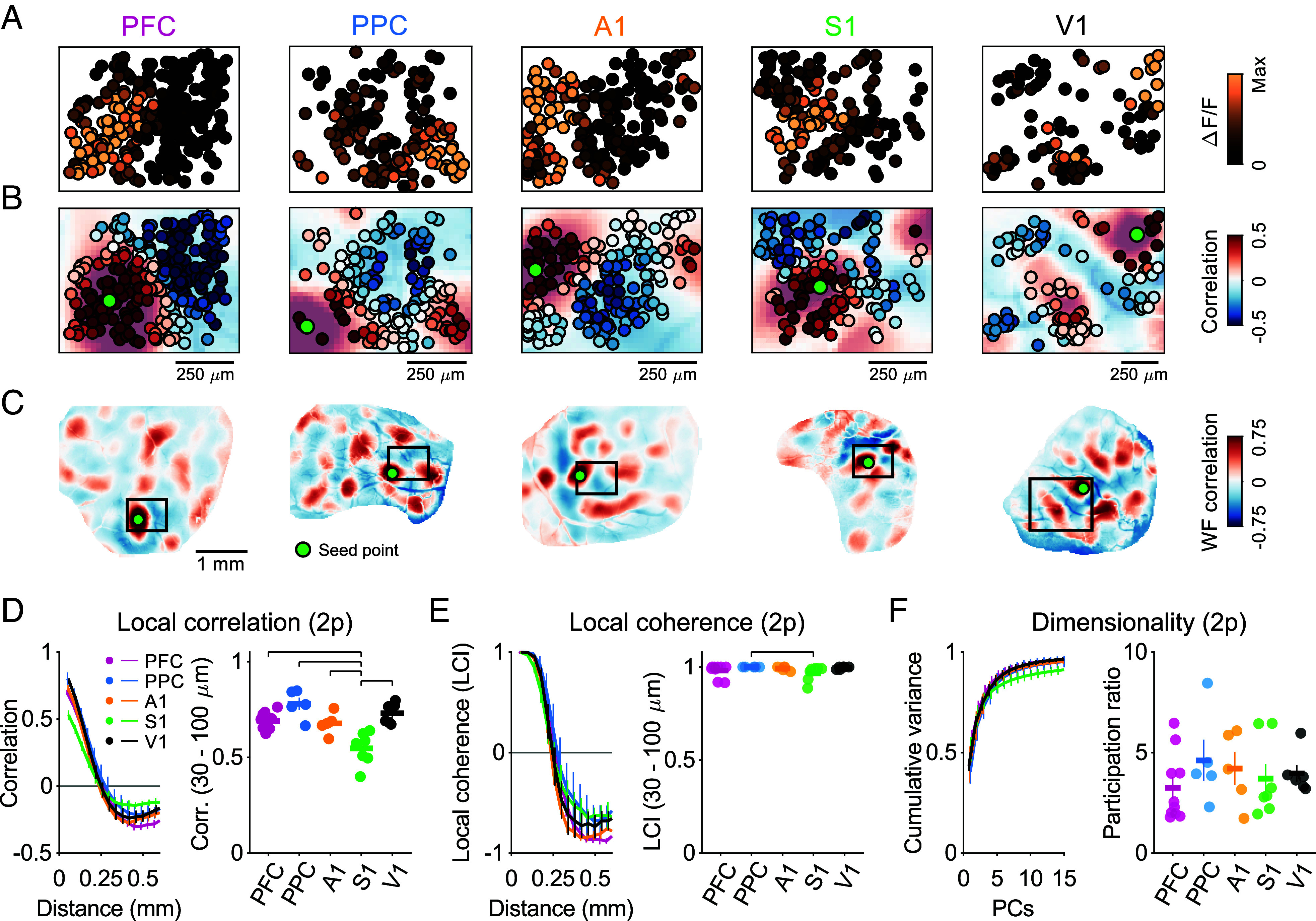
Spontaneous activity shows strong local organization with cellular resolution across the cortex. (*A*) Modular organization of spontaneous activity in layer 2/3 neurons is evident in individual events. (*B*) Activity across events is locally correlated and modular. Correlations for individual neurons are shown relative to seed neuron (in green) and are overlaid on correlations from widefield imaging in the same animal (see below), showing a strong correspondence between millimeter-scale networks in widefield imaging and local organization at the cellular level. (*C*) Widefield correlation patterns for seed point matching location of seed neuron shown in (*B*). Box indicates 2-photon FOV shown in (*A* and *B*). (*D*, *Left*) The amplitude of correlations between neurons shows a similar pattern with distance across areas. (*Right*) Nearby correlations (30 to 100 µm) are strong in all cases. Correlations in S1 are significantly weaker than other areas. (*E*) LCI of spontaneous correlations shows highly organized functional networks. (*Left*) The reversal from positive to negative correlations occurs at a similar distance in all cortical areas. (*Right*) Coherence is nearly uniform (near 1) for nearby populations of neurons (30 to 100 µm). (*F*) Dimensionality of spontaneous activity within local populations of neurons is moderately low and does not vary significantly across areas. (*Left*) Cumulative variance explained and (*Right*) participation ratio for populations of 50 neurons in each area. Horizontal lines in (*D* and *E*) indicate significant post hoc pairwise comparisons. Error bars ± SEM.

The presence of widespread modular activity at millimeter scale throughout the cortex suggests a degree of coordinated activity among local populations of neurons. However, given that widefield imaging lacks the spatial resolution to identify individual neurons and instead reports the pooled contributions of local populations, it is possible that the functional modules we observe actually reflect a more heterogeneous local structure. In such a case, an active module might result from the activation of only a sub-population of neurons in a local region, potentially intermixed in a salt-and-pepper fashion with non-participating inactive neurons. In order to address this, we performed 2-photon imaging of spontaneous activity in layer 2/3 neurons in all cortical areas. We observed that spontaneous events in layer 2/3 neurons in all areas exhibited a clear tendency for nearby neurons to be co-active, with events showing clear spatially contiguous patches of active neurons extending several hundred microns, largely without intermixed inactive neurons (Fig. 3*A* and *SI Appendix*, Fig. S7). The patterns of co-active neurons exhibited strong local pairwise correlations across events, which decreased as a function of distance in all areas (Fig. 3 *B* and *D*). Correlations between nearby neurons were statistically significant in all areas (Fig. 3*D*; 30 to 100 μm: (mean ± SEM) PFC: 0.67 ± 0.02 (n = 10 FOV, 6 animals, 589 total events); PPC: 0.77 ± 0.05 (n = 5 FOV, 4 animals, 423 events); A1: 0.67 ± 0.03 (n = 5 FOV, 4 animals, 419 events); S1: 0.49 ± 0.04 (n = 7 FOV, 4 animals, 645 events); V1: 0.73 ± 0.03 (n = 6 FOV, 5 animals, 508 events), 33 of 33 FOVs significant vs. shuffle control, event numbers in *SI Appendix*, Table S2). Local correlations between neurons in S1, while highly significant vs. control, were slightly but significantly weaker than those in other areas (KW: H(4) = 18.18, *P* = 0.001; post hoc: PFC vs. S1: *P* = 0.0114, PPC vs. S1: *P* = 0.0001, A1 vs. S1: *P* = 0.0208, V1 vs. S1: *P* = 0.0003, *SI Appendix*, Table S5). This strong local organization in correlated functional activity was readily apparent in the local coherence index (LCI), which reflects the sign of correlations (positive vs. negative) irrespective of strength (*Materials and Methods*). LCI was highly similar across areas over distance, showing a similar spacing of the transition from locally positive to more distant negative correlations, consistent with the similar wavelength of modular activity seen above in widefield data and reflecting a similar size of functional modules across areas (Fig. 3*E*; (mean ± SEM) PFC: 0.98 ± 0.01 (n = 10 FOVs); PPC: 1.00 ± 0.00 (n = 5); A1: 0.99 ± 0.01 (n = 5); S1: 0.97 ± 0.02 (n = 7); V1: 1.00 ± 0.00 (n = 6); KW: H(4) = 10.19, *P* = 0.037; post hoc: PPC vs. S1 *P* = 0.0168, *SI Appendix*, Table S6). Moreover, we found that all areas exhibited a moderately low dimensionality within local populations of individual neurons, consistent with the presence of strong local neural correlations (Fig. 3*F*; dimensionality for subsets of 50 neurons (*Materials and Methods*): (mean ± SEM) PFC: 3.25 ± 0.53 (n = 10 FOVs); PPC: 4.61 ± 1.03 (n = 5); A1: 4.21 ± 0.82 (n = 5); S1: 3.71 ± 0.73 (n = 7); V1: 3.97 ± 0.41 (n = 6); KW: H(4) = 2.71, *P* = 0.607).

To assess whether the spatial layout of the millimeter-scale networks observed with widefield imaging is reflected at the cellular level, we aligned our 2-photon FOVs to the widefield images collected in the same animal. In all cases, we found that the patterns of correlations showed a strong correspondence to those seen in widefield (Fig. 3 *B* and *C*). These results indicate that the modular patterns of activity observed with widefield imaging in all cortical areas reflect the locally clustered activity of layer 2/3 neurons, consistent with prior results in V1 (16, 20). Collectively, these results demonstrate that highly coherent modular organization is a common motif of spontaneous activity among local populations of layer 2/3 neurons in the developing cortex across both sensory and association cortices.

## Discussion

By examining spontaneous activity early in cortical development, we show that diverse cortical areas including both sensory and association regions share a surprisingly similar network activity structure: A distributed and modular organization of neural activity that reveals millimeter-scale functionally correlated networks. In fact, we find that this common structure of spontaneous activity at both the cellular and columnar level across these diverse areas is highly similar to that previously found in V1 in early development, where such modular structure is predictive of future sensory representations (20) and has long been known as a hallmark of functional activity (11, 12). These results argue strongly that the diverse representations found across brain areas in the mature cortex emerge during development from an initially common functional organization that is shared across the cortex—a pluripotent cortical substrate—and suggests the developmental origin of these diverse representations adheres to common design principles.

This idea has its roots in the “protocortex” hypothesis (41), holding that the early cortex is relatively homogeneous, with functional specialization emerging over development in response to area-specific inputs. While much subsequent work has shown that areal specialization involves a complex interplay of genetic and activity-dependent mechanisms (reviewed in refs. 5 and 6), the concept of a canonical cortical organization with a modular or columnar structure has remained powerful, although the structure of this organization may vary, ranging from mini-columns of a few cells to the so-called macro-columns seen in V1 orientation maps (21). The modular activity patterns we observe across areas are consistent with this latter spatial scale, arguing that such features are present across many areas in the developing cortex. Additionally, our results extend this idea by showing that within these diverse areas modular activity is functionally organized into distributed millimeter-scale correlated networks, suggesting the presence of a shared large-scale network structure that is common across cortical regions.

We performed our experiments under light isoflurane anesthesia. Such an approach allowed us to obtain stable and high-quality imaging from very young animals, at a timepoint when the training and habituation required for awake head-fixed recordings is challenging. Importantly, prior work in V1 has demonstrated that while anesthesia impacts the rate of spontaneous events, it does not impact their modular spatial structure or their spatial correlations, both of which were indistinguishable between awake and anesthetized recordings (20). These prior results argue that the correlation structure of spontaneous activity reflects features of the cortical network organization rather than state-dependent motifs.

Our finding of a quantitatively similar modular structure across areas raises the possibility that the circuit-level mechanisms generating this functional organization may likewise be common across regions. In V1, the columnar organization has been attributed to feed-forward influences such as the organization of retinal ganglion cell mosaics (42, 43) or orderly inputs driven by retinal waves (44). However, our results challenge these explanations, as these features are specific to the visual pathway and therefore cannot readily account for modular organization in non-visual areas, as the transfer of modular patterns between areas would require isotropic and retinotopically mapped projections from V1, which are not known to exist (45). Although it is possible that the inputs to diverse areas such as PFC and S1 each have their own independent organizing structure, perhaps involving thalamic waves in a manner analogous to that of the retina for V1, a simpler explanation could be that modular functional activity is generated independently and locally within each cortical area. This hypothesis is supported by data from V1 showing that modular spontaneous activity in this region appears to be generated intracortically (20, 35), potentially through mechanisms of self-organization (34, 46–51). Future studies will be required to determine whether such intracortical mechanisms operate locally in diverse cortical areas during early development.

If this is the case, then the presence of modular organization in a cortical area would result from the structure of the cortical circuits themselves, and not simply reflect the organization imposed by external inputs. This contrast is highlighted in classic experiments showing that A1 can be re-wired with visual input to generate a modular map of orientation preference (52, 53), experiments which were originally interpreted as evidence for an organizing role of feed-forward visual input (54). However, our results instead argue that the auditory cortex itself already possesses a modular functional organization in early development prior to any re-wired visual inputs. This then raises an intriguing possibility: that universal and generic intracortical circuitry gives rise to distributed modular networks through self-organizing mechanisms that then serve as a pluripotent cortical substrate for feed-forward inputs to generate area-specific modular neural representations.

In such a regime, the functional specificity found across areas in the mature brain may emerge later, in response to area-specific influences acting upon an initially common modular organization. Intriguingly, while highly similar to other areas in most respects, S1 did show statistically significant differences in several measures, including module amplitude and local correlation strength, raising the possibility that such area-specific impacts might already be influencing S1 at these timepoints. Sensory evoked representations emerge in S1 prior to other sensory areas (8), suggesting an earlier time course of maturation. Indeed, well-organized peripherally driven cortical responses can be elicited as early as E18.5 in mice (9). Future studies looking at earlier developmental timepoints will be able to determine whether modular spontaneous activity in S1 more closely matches other cortical areas, or rather exhibits functional differences throughout development.

The large-scale correlations seen in our results are based on calcium imaging, which has relatively slow temporal resolution, leaving open the question of faster temporal dynamics in the developing cortex. Multiple studies from a range of species have utilized voltage-sensitive dye (VSD) imaging or multi-electrode arrays to reveal fast propagating waves of activity across the cortical surface (reviewed in refs. 55 and 56). Such waves have been observed across a range of cortical areas, including (but not limited to) V1, MT, somatosensory, and motor cortex (e.g., refs. 57–62), and have been linked to both perception (60) and motor output (63). In some cases, the propagation of these waves appear to be restricted to single cortical areas [e.g. V1 vs. V2 in awake monkeys (64)], whereas in other situations (such as in rodents under anesthesia) activity appears to spread across area boundaries (57). Notably, the propagation of activity in V1 appears to align with the columnar arrangement of orientation preference (58, 59), suggesting that such propagating activity may not always exhibit a single continuous wave front, but rather can reflect modular organization. These fast propagating waves seen in VSD and local field potentials largely reflect subthreshold depolarization that can promote spiking activity, thereby potentially contributing to the modular organization we observe with calcium imaging. As such propagating activity may play a role in the functional maturation of cortical circuits (reviewed in ref. 65), future experiments will examine the relationship of fast propagating activity to the modular organization of developing cortex.

Is modular activity a general principle of cortical organization? Modular structures have been shown to be potentially advantageous in a number of contexts, both from a wire-minimization perspective (66), as well as conveying advantages in information processing including increased robustness and adaptability (67). For example, in the primary visual cortex, a modular representation of edge orientation may facilitate the detection of object contours from images (68). Additionally, previous work in V1 has shown that the modular structure of spontaneous activity in early development serves as a precursor to these mature representations (20). Thus, our results suggest the possibility that the modular networks we observe in cortical areas such as PFC during early development may likewise go on to encode modular representations of features in the mature cortex, an idea supported by prior work showing spatially clustered afferent projections in PFC (32, 69). Additionally, the presence of clustered patchy horizontal projections in many regions of the mature cortex is also consistent with the widespread presence of distributed modular organization (70, 71). While a functional characterization of these mature cortical networks will require future study, our results highlight the ability of spontaneous activity to reveal features of network organization even without precisely designed stimulation paradigms, thereby providing a powerful tool to investigate areas with unknown or complex neural representations, such as higher-order association cortices. Furthermore, by examining spontaneous activity across cortical regions in more mature animals, it will be possible to determine whether the common modular organization we observe throughout the early developing cortex is maintained across development, or rather undergoes area-specific changes, for instance through sparsification of neural activity patterns.

What role might a common modular functional organization play in the early cortex? Such organization could allow the establishment of local neural representations coupled through long-range projections in an efficient coarse (module) to fine (cellular) manner over development. In this way, the correlated spontaneous activity we observe throughout the cortex could serve as an instructive signal, driving Hebbian plasticity both within and among locally clustered circuits. Such a role is supported by the finding in V1 that early modular correlations in spontaneous activity are predictive of structural attributes of future sensory representations (20). Notably, such a structure could also facilitate the formation of cross-area connectivity: As the dimensionality of spontaneous activity is roughly matched across brain areas, plasticity mechanisms would be readily able to operate at equivalent scales throughout the cortex, promoting cross-area communication and the establishment of distributed, multimodal and cortex-wide representations early in development. In this way, our findings suggest that rather than only potentially acting to optimize coding in the mature cortex, a modular organization may also serve as a developmental guidepost for circuit assembly that is maintained in some cortical areas but perhaps not in others.

## Materials and Methods

### Data Collection.

#### Animals.

All experimental procedures were approved by the University of Minnesota Institutional Animal Care and Use Committee and were performed in accordance with guidelines from the US NIH. We obtained eight male and female ferret kits from Marshall Farms and housed them with jills on a 16-h light/8-h dark cycle. No statistical methods were used to predetermine sample sizes, but our sample sizes are similar to those reported in previous publications.

#### Viral injection.

Viral injections were performed as previously described (72). Briefly, we expressed GCaMP6s (33) in neurons by microinjecting AAV1.hSyn.GCaMP6s.WPRE.SV40 (Addgene) into layer 2/3 of targeted cortical areas at P13-15, 7 to 10 d before imaging. Anesthesia was induced with isoflurane (4 to 5%) and maintained with isoflurane (1 to 1.5%). Glycopyrrolate (0.01 mg/kg) and bupivacaine/lidocaine (1:1 mixture) were both administered, and animal temperature was maintained at approximately 37 °C with a water pump heat therapy pad (Adroit Medical HTP-1500, Parkland Scientific). Animals were also mechanically ventilated and both heart rate and end-tidal CO2 were monitored throughout the surgery (Digicare LifeWindow). Using aseptic surgical technique, skin and muscle overlying target areas were retracted, and a small burr hole was made with a handheld drill (Fordom Electric Co.). Approximately 1 µL of virus contained in a pulled-glass pipette was pressure injected into the cortex at two depths (~200 µm and 400 µm below the surface) over 20 min using a Nanoject-III (World Precision Instruments). The craniotomy was sealed and the skin sutured closed.

Targets for different cortical areas were as follows:

V1: ~6 to 8 mm lateral from midline, ~1 to 2 mm anterior to the Sinus
PPC: ~1 to 2 mm lateral from midline, ~4 mm posterior to Bregma
A1: ~7 to 9 mm lateral to midline, ~3 mm posterior to Bregma
S1: ~2 to 3 mm lateral to midline, ~ 1 mm anterior to Bregma
PFC: ~1 to 2 mm lateral to midline, ~7 to 8 mm anterior to Bregma

We injected virus into and imaged 1 to 5 areas per animal. For a full list of animal ages and targets, See *SI Appendix*, Fig. S8.

#### Cranial window surgery.

On the day of experimental imaging, ferrets aged P21-24 were anesthetized with 3 to 4% isoflurane and atropine (0.2 mg/kg) or glycopyrrolate (0.01 mg/kg) was administered. Animals were placed on a feedback-controlled heating pad to maintain an internal temperature of 37 °C. Animals were intubated and ventilated. Isoflurane was delivered between 1 and 2% throughout the surgical procedure to maintain a surgical plane of anesthesia. An intraperitoneal or intravenous catheter was placed to deliver fluids. Electrocardiogram, end-tidal CO2, and internal temperature were continuously monitored during the procedure and subsequent imaging session. The scalp was retracted and a custom titanium headmount adhered to the skull using C&B Metabond (Parkell). A 6- to 7-mm craniotomy was performed over areas of viral expression and the dura was retracted to reveal the cortex. Cover glass (round, #1.5 thickness, Electron Microscopy Sciences) adhered to the bottom of a custom titanium or 3-D printed plastic insert was placed onto the brain to gently compress the underlying cortex and dampen biological motion during imaging. Upon completion of the surgical procedure, isoflurane was gradually reduced (0.6 to 1.0%) and then vecuronium bromide (2 mg/kg/h) was delivered to reduce motion and prevent spontaneous respiration.

#### Widefield epifluorescence and two-photon imaging.

Spontaneous activity was recorded in a quiet darkened room for 10 to 40 min. Widefield epifluorescence imaging was performed with a Zyla 5.5 sCMOS camera (Andor) controlled by MicroManager software (73). Images were acquired at 15 Hz with 4 × 4 binning to yield 640 × 540 pixels. Two-photon imaging was performed on a Neurolabware microscope with Scanbox software (Los Angeles, California, USA) using either a 16× (Nikon) or 25× (Olympus) objective. Excitation was provided by an InSight X3 femtosecond laser (Spectra Physics) at 940 nm. Images were acquired at 512 × 768 pixels at 30 Hz.

#### Histology.

Following imaging, animals were euthanized with 5% isoflurane and pentobarbital. Animals were perfused with heparinized saline solution followed by 4% paraformaldehyde, then the brains were removed and kept for histology. Viral expression of GCaMP was documented in the intact brain with appropriate excitation and emission filters. Images of expression were aligned to a common coordinate system using prominent brain features (sulci, fissures, and external edges of the brain). Areas of expression from each brain were outlined manually in Matlab and are shown in *SI Appendix*, Fig. S8.

### Analysis Methods.

#### Widefield data pre-processing.

Widefield data pre-processing, event extraction, and calculation of spontaneous correlations were performed largely as described (20) for all imaged areas. Briefly, to correct for mild brain movement during imaging, we registered each imaging frame by maximizing phase correlation to a common reference frame. A region of interest (ROI) was manually drawn around the cortical area with high and robust spontaneous activity. ROIs were also drawn to remove any artifacts or debris in the visible FOV. The baseline fluorescence (F_0_) for each pixel was obtained by applying a median filter to the raw fluorescence trace with a window between 10 and 23 s. Filter width was chosen for each imaging session individually, such that the baseline followed faithfully the slow trend of the fluorescence activity. The baseline corrected activity was calculated as

[1] $$\left(F-F_{0}\right)/F_{0}=\Delta F/F_{0}.$$

#### Event detection.

To detect spontaneously active events, we first determined active pixels on each frame using a pixel-wise threshold set to 3 SD above each pixel’s mean value across time. Active pixels not part of a contiguous active region of at least 0.01mm^2^ were considered “inactive” for the purpose of event detection in order to minimize detecting noise as “active” pixels. Active frames were taken as frames with a spatially extended pattern of activity (>40% of pixels were active). Consecutive active frames were combined into a single event starting with the first high activity frame and then either ending with the last high activity frame or, if present, an activity frame defining a local minimum in the fluorescence activity. In order to assess the spatial pattern of an event, we extracted the maximally active frame for each event (the “event frame”), which was defined as the frame with the highest activity averaged across the ROI. Temporal autocorrelation was computed using all frames in the imaging session, including event and non-event frames, for lags up to 10 s (155 frames).

#### Calculation of correlation patterns.

To assess the spatial correlation structure of spontaneous or evoked activity, we applied a Gaussian spatial band-pass filter (with SD of Gaussian filter kernel *s*_high_ = 195 µm, s_low_ = 26 to 41 µm) to each event frame and down-sampled it to 160 × 135 pixels. The resulting patterns, named *A_i_* in the following, where i = 1,…,N, were used to compute the spontaneous correlation patterns as the pairwise Pearson’s correlation between all locations ***x*** within the ROI and the seed point ***s***

[2] $$\begin{array}{c} C(x,s)=\frac{1}{N}\frac{\Sigma_{i=1}^{N}(A_{i}(x)-<A_{i}(x)<)\;(A_{i}(s)-<A_{i}(s)>)}{\sigma_{x}\sigma_{s}} \end{array}$$

Here, the brackets < > denote the average over all *N* patterns and *σ****_x_*** denotes the SD of *A* over all *N* patterns at location ***x***.

Note that the spatial structure of spontaneous activity was already evident without filtering (*SI Appendix*, Fig. S1), and our results did not sensitively depend on filtering.

#### Shuffled control ensemble and surrogate correlation patterns.

To evaluate the statistical significance of quantities characterizing the correlation patterns observed during spontaneous activity, we compared the real ensemble of spontaneous activity patterns from a given experiment with a control ensemble, obtained by eliminating most of the spatial relationships between the patterns. To this end, all activity patterns were randomly rotated (rotation angle drawn from a uniform distribution between 0° and 360° with a step size of 10°) and reflected (with probability 0.5, independently at the x- and y-axes at the center of the ROI), resulting in an equally large control ensemble with similar statistical properties, but little systematic interrelation between patterns. Surrogate correlation patterns were then computed from these ensembles as described above.

#### Dimensionality of spontaneous activity.

We estimated the cross-validated dimensionality *d*_eff_ of the subspace spanned by spontaneous activity patterns (see ref. 40). First, we randomly divided the activity patterns into two non-overlapping subsets X_1_ and X_2_, and then performed PCA on X_1_ to find the axis spanned by it. Next, we projected X_2_ onto these PCs to estimate the variance explained by each PC, λ_i_. Last, dimensionality was calculated as (39):

[3] $$d_{eff}=\frac{{\left(\sum_{i=1}^{N}{\text{λ}}_{i}\right)}^{2}}{\sum_{i=1}^{N}{\text{λ}}_{i}^{2}}.$$

#### Spatial range of correlations.

To assess the strength of spontaneous correlations over distance (Fig. 2), we identified the local maxima (minimum separation between maxima 800 µm) in the correlation pattern for each seed point. The amplitude of correlations at these maxima was then pooled across all seed points. For this analysis, we standardized the number of events across animals and areas by calculating correlation patterns on N = 100 events randomly subsampled from all recorded events. To assess the statistical significance of long-range correlations ~2 mm from the seed point, we compared the median correlation strength for maxima located 1.8 to 2.2 mm away against a distribution obtained from 100 surrogate correlation patterns. For individual animals, the *P*-value was taken as the fraction of correlation strength values from surrogate data greater than or equal to the median correlation strength for real correlation patterns. The ROI for one A1 FOV was too small to compute a surrogate dataset at 2 mm, and was excluded from this analysis.

As an alternate approach to determine the spatial range of correlations, we computed the variance of pixelwise correlation values located at a given distance from a seed point (74). Closer to the seed point (e.g. gray shaded region in *SI Appendix*, Fig. S5*A*) correlations will have both strongly positive and strongly negative values, leading to a high variance. In contrast, further away from the seed point (e.g. magenta shaded region in *SI Appendix*, Fig. S5*A*) correlations will be closer to zero and exhibit reduced variance. We computed the variance within a ring of increasing radius (0.2 mm bins, from 0.8 to 2.2 mm from seed point). To control for the finite number of events in our dataset, we also computed the variance for surrogate correlation patterns (see above) generated for each experiment using a matched number of patterns (*SI Appendix*, Fig. S5 *C*–*E*). Subtracting the control variance for each experiment allows for comparison across experiments with varying numbers of events (*SI Appendix*, Fig. S5 *F* and *G*).

#### Modularity and wavelength estimation.

To estimate the wavelength of individual calcium events, we calculated the 1-D radial average of the spatial autocorrelation of the band-pass-filtered activity pattern (*SI Appendix*, Fig. S3 *A*–*C*). The wavelength of the event was taken as twice the distance to the first minimum from the origin. Modularity is a measure of the regularity of the spatial arrangement of activity domains within the pattern. The modularity of each event was calculated as the absolute difference in amplitude between the first minimum and the subsequent maximum of the 1-D radial averaged autocorrelation.

To determine whether the modularity observed during spontaneous events was statistically significant, we compared it to a distribution of modularity values for inactive frames used as the control. Control frames were drawn from the bottom 10% of frames lacking an identified spontaneous event (see above) based on mean activity within the ROI. For each experiment, we obtained 100 sets of control frames containing an event-matched number of frames and calculated the median modularity across these frames, generating a distribution of 100 control modularity values. This was then compared to the median modularity across spontaneous events to obtain a *P*-value.

#### Module amplitude.

We defined the module amplitude of an individual widefield event as the average amplitude (in ΔF/F, prior to spatial filtering) of the module peaks divided by the background activity. Peaks in activity for each event were first obtained by using the FastPeakFind.m function in Matlab (75). Background activity was taken as the median amplitude (in ΔF/F) of activity in locations ½ wavelength from the peak. Here, the average wavelength across all events was used for each FOV. The module amplitude of an event was taken as the average amplitude across all peaks in the event.

#### 2-photon data pre-processing.

2-photon images were registered to remove any motion artifacts using Scanbox and Matlab. We selected cellular ROIs in our 2-photon data manually using the cell magic wand tool (76) in ImageJ, and imported these ROIs into Matlab via MIJ (77). Fluorescence traces were then extracted from these ROIs and neuropil subtracted:

[4] $$F_{\text{cell}}{=F}_{\text{raw}}{-\alpha *F}_{\text{neuropil}},$$

where α = 0.4 and F_neuropil_ was taken as the average signal in a 20-μm window around the neuron excluding other labeled cells. We obtained the F_0_ value for these calcium signals by using a median filter with a 60-s window. The baseline corrected activity was calculated as (*F−F*_0_)/*F_0_* = Δ*F*/*F*_0_. In comparisons, our results were qualitatively similar with and without neuropil subtraction. Data were smoothed with a median filter over seven frames. In several cases, we recorded more than one 2-photon FOV in a single cortical area of a single ferret (at a different x-y location within the area of expression). 2-photon FOVs were aligned to widefield imaging in the same animal using surface and penetrating blood vessels as reference points.

#### 2-p spontaneous event detection.

For a given frame, active neurons were identified as cells with Δ*F*/*F*_0_ 2 SD above their mean. Frames with over 5% of neurons active were taken as spontaneous events. Consecutive active frames were combined into a single event starting with the first high activity frame and then either ending with the last high activity frame or, if present, an activity frame defining a local minimum in the number of active cells. We extracted a single event frame as the frame with the highest average signal across all cells during the event. The activity of cells within these event frames was z-scored across the frame, and pairwise correlations across all cells were calculated. Correlations were compared against a distribution of 100 surrogate correlation patterns obtained by first shuffling activity within each cell across all events. For individual animals, the *P*-value was taken as the fraction of median correlation strength from surrogate data greater than or equal to the median correlation strength for real correlation patterns. The LCI was calculated for each seed neuron as (N_pos_ − N_neg_)/(N_pos_ + N_neg_), where N_pos_ (N_neg_) is the number of positively (negatively) correlated cells within an annulus of increasing radius, excluding cells with correlations −0.01< × <0.01. Values range from 1 (all cells within annulus positively correlated) to −1, and were averaged over all seed points in a FOV.

#### Dimensionality of cellular activity.

Similar to EPI-dimensionality, cross-validation was used to estimate PCs and explained variances. We drew 100 random samples of 50 cells and 100 event frames from each FOV to control for the sample size. Dimensionality was calculated using Eq. **3**, and we averaged over these 100 samples to obtain the final dimensionality value.

### Statistical Methods.

Nonparametric statistical analyses were used throughout the study. All tests were two-sided unless otherwise noted. Comparisons across areas were performed using a KW test. Significant across-group differences (alpha = 0.05) were followed by pairwise post hoc Conover-Iman tests with Holm’s correction for multiple comparisons. Post hoc tests were implemented with the conover.test package in R (78). All pairwise post hoc comparisons are listed in *SI Appendix*, Tables S3–S6, only post hoc comparisons with *P* < 0.05 are also listed in main text, for space considerations. We used alpha = 0.05 unless otherwise stated.

Data analysis was performed in Matlab (Mathworks), Python, and R (v3.6.0; R Core Team 2019).

## Supplementary Material

Appendix 01 (PDF)

Movie S1. Spontaneous activity in PFC.

Movie S2. Spontaneous activity in PPC.

Movie S3. Spontaneous activity in A1.

Movie S4. Spontaneous activity in S1.

Movie S5. Spontaneous activity in V1.

## Data Availability

Data and code used to produce all figures is available at https://github.com/SmithNeuroLab/multiarea (79). All study data are included in the article and/or SI Appendix.
