# Supplementary material for: Common modular architecture across diverse cortical areas in early development
Source: Proc Natl Acad Sci U S A. 2024 Mar 6;121(11):e2313743121. doi: 10.1073/pnas.2313743121 (PMC10945769; doi:10.1073/pnas.2313743121)
Supplement: Supplementary file 1 — Appendix 01 (PDF) [file pnas.2313743121.sapp.pdf]

PNAS

Supporting Information for
Common modular architecture across diverse cortical areas in early development

Nathaniel J. Powell, Bettina Hein, Deyue Kong, Jonas Elpelt, Haleigh N. Mulholland, Matthias Kaschube, Gordon B. Smith

Corresponding author: Gordon B. Smith
Email: gbsmith@umn.edu

This PDF file includes:

Figures S1 to S8
Tables S1 to S6
Legends for Movies S1 to S5

Other supporting materials for this manuscript include the following:

Movies S1 to S5

Figure S1

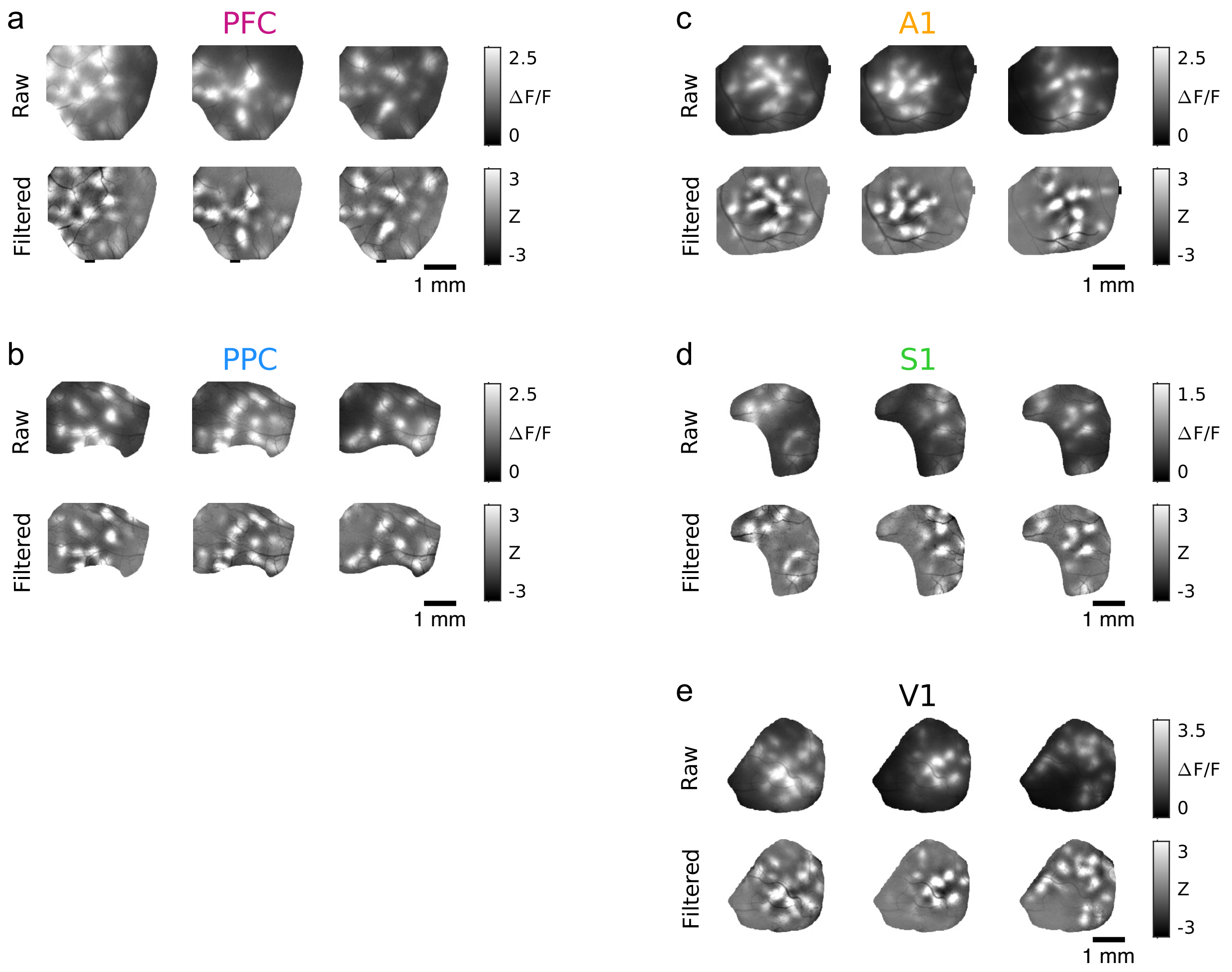

Fig. S1. Modular structure of spontaneous activity is readily apparent without spatial filtering. a. Example spontaneous events in PFC. *Top row*: events shown without spatial band-pass filtering exhibit clear modular organization. *Bottom row:* same events after band-pass filtering. **b-e.** Same for PPC, A1, S1, and V1, respectively.

Figure S2

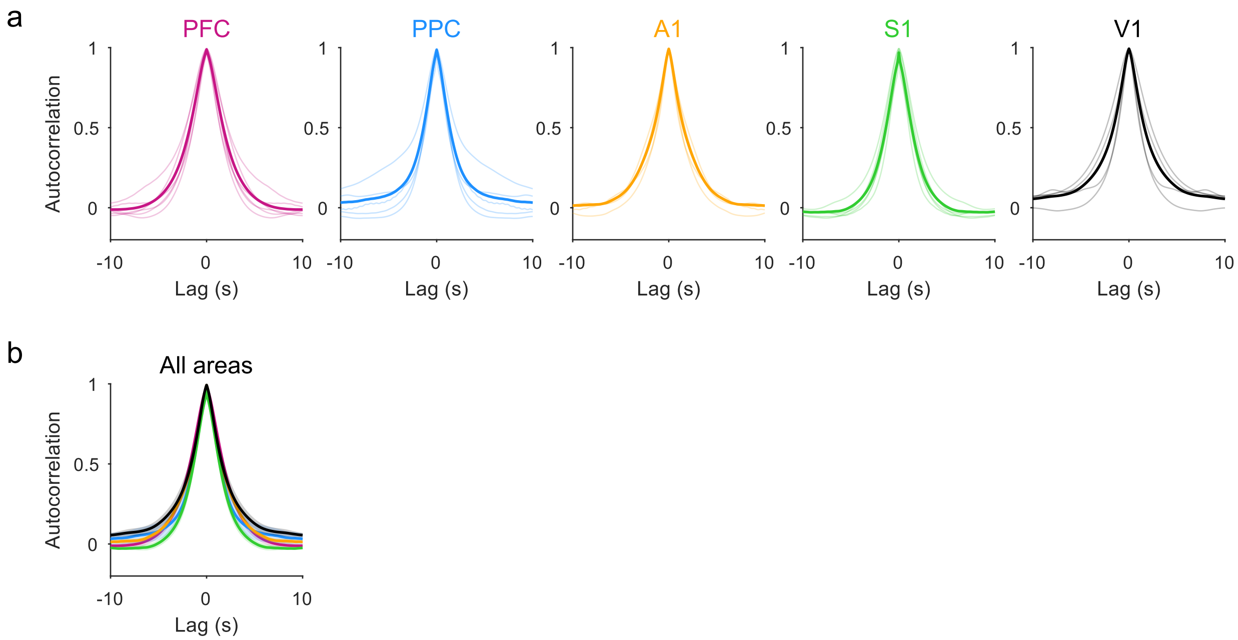

Fig. S2: Temporal autocorrelation of spontaneous activity. a. Temporal autocorrelation for all frames for each cortical area. Thin lines show individual animals, thick line shows mean across animals. **b**. Autocorrelations are similar across areas. Shaded area indicates mean ± SEM across animals within each area.

Figure S3

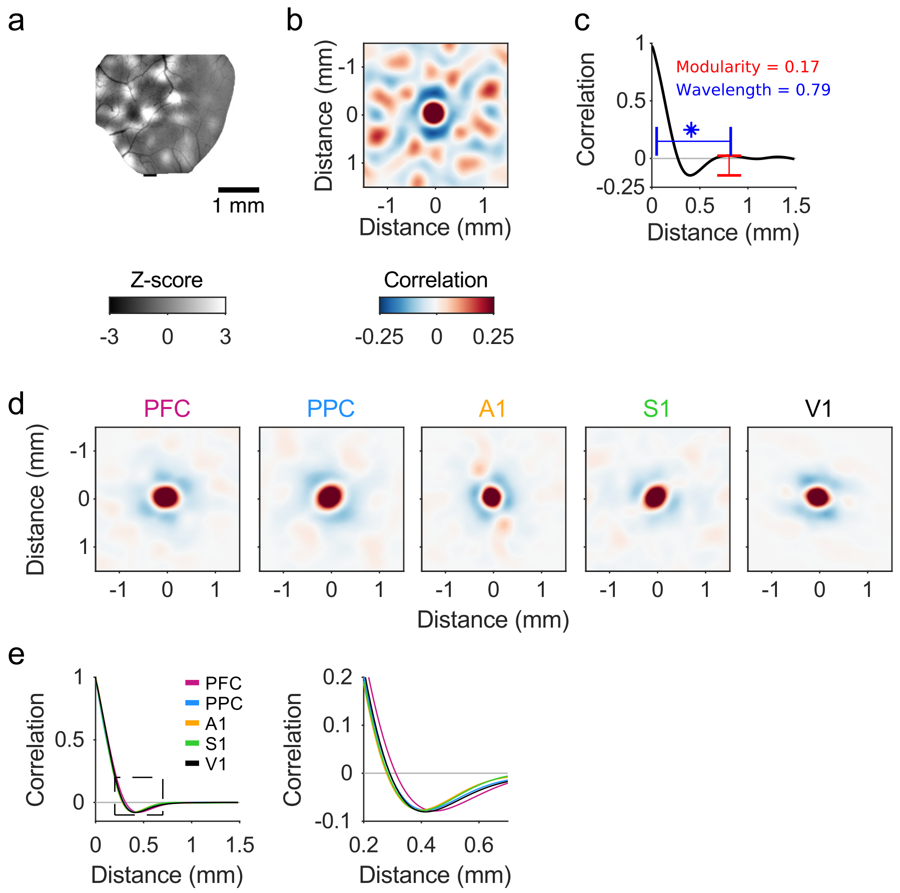

Fig. S3: Determination of event modularity and wavelength. The modularity and wavelength of events are calculated from the radial average of the spatial autocorrelation of individual spontaneous events. **a.** Example spontaneous event. **b.** 2-D spatial autocorrelation. **c.** 1-D radial average of autocorrelation. Modularity is measured as the height of the first peak after the origin above the first trough. Wavelength is taken as twice the distance of the first trough (indicated by blue star). **d.** Autocorrelation patterns averaged across events for representative FOVs for each area show similar spatial structure indicating similar domain size and spacing. **e.** 1-D autocorrelations averaged across all animals. *Right:* expanded view of region shown in box to left.

Figure S4

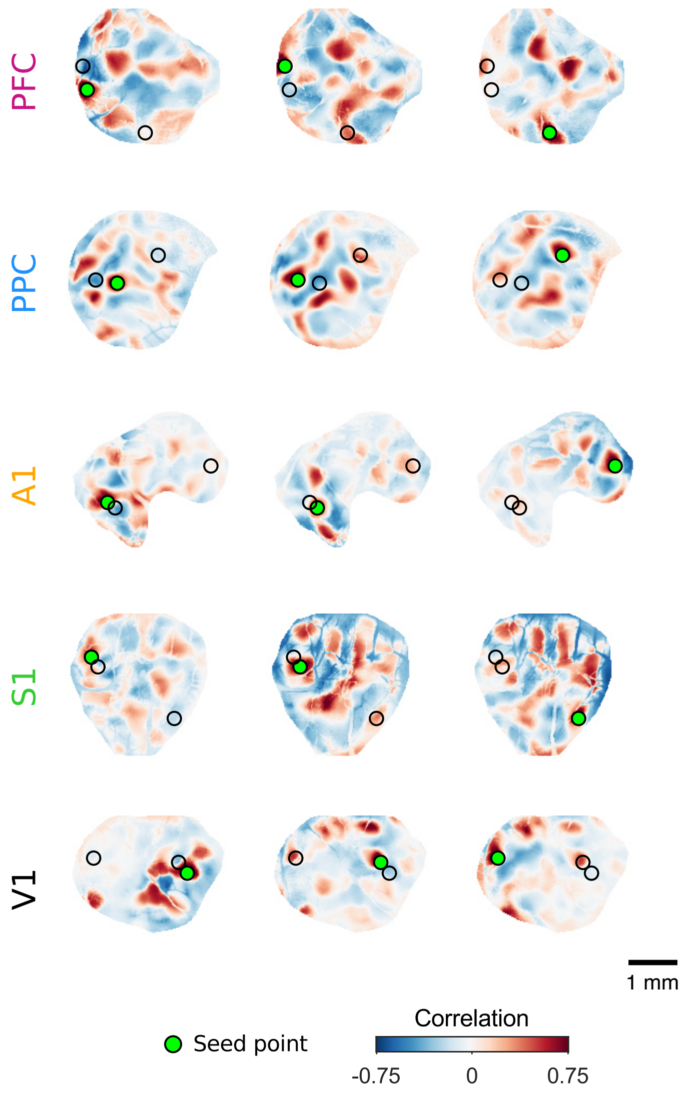

Fig. S4. Multiple distributed and modular functional networks exist within each cortical area. For each area, additional examples of correlation patterns for differing seed points reveal the presence of multiple correlated networks within each imaged region. Correlation patterns can vary greatly for nearby seed points (e.g. *left, middle* columns). Distant seed points can also participate in highly similar long-range correlated networks (*middle, right* columns). Seed point for displayed correlation pattern is shown in green, open circles show locations for seed points displayed in adjacent columns.

Figure S5

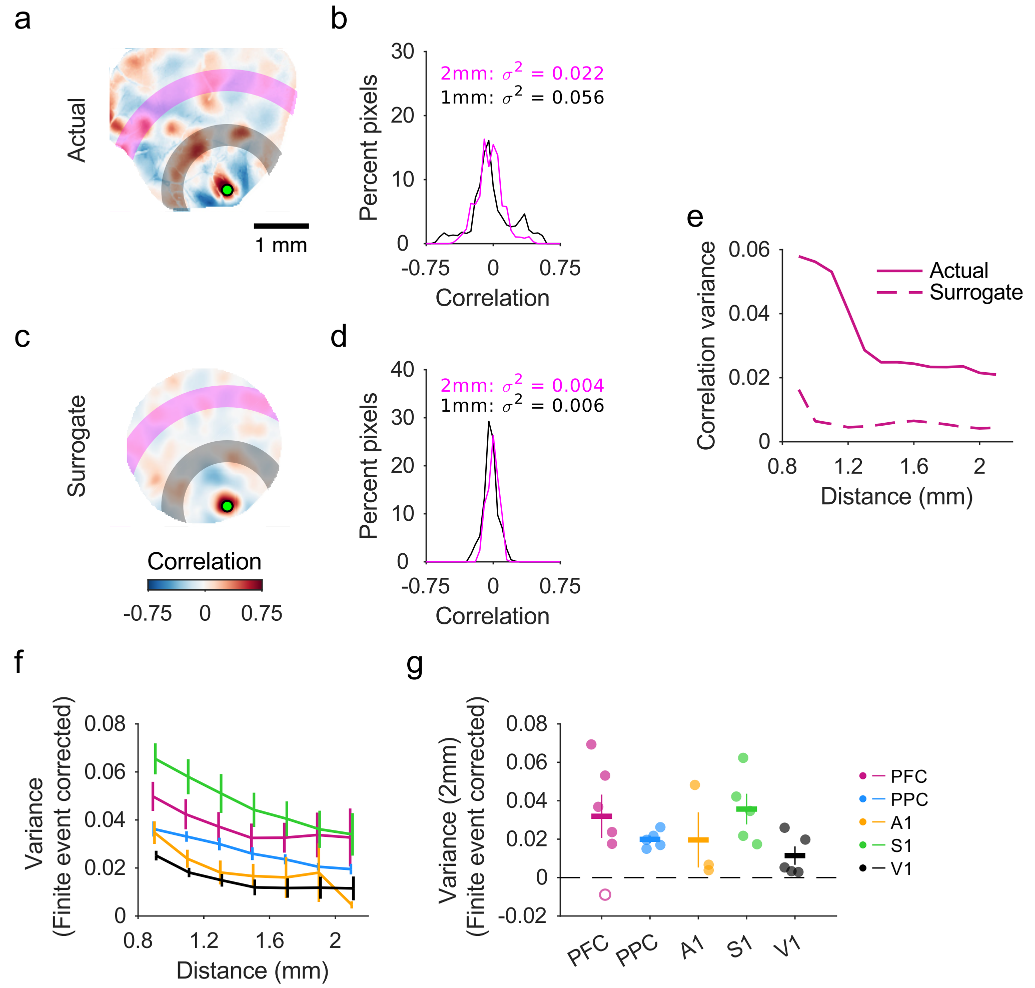

Fig. S5. Assessment of long-range correlations in spontaneous activity through correlation variance. **a,b.** Correlation strength was measured by computing the variance of the distribution of correlation values within bins of increasing distance from the seed point. Correlation pattern with distance bins is shown in (a) and the distribution of correlation values within these bins in (b). Regions of strong correlations have both strongly positive and strongly negative values within the bin, leading to a high variance (e.g. near a seed point, black line). Weak correlations (e.g. further from a seed point, magenta) will be closer to zero and exhibit reduced variance. **c,d.** Same as for (a,b) but for a surrogate correlation pattern computed for an equal number of patterns as in (a) (see Methods). Distributions of correlation values are narrow, and vary little with distance. **e.** Plot of correlation variance as a function of distance from the seed point for the 'actual' correlation pattern (a) and the surrogate pattern (c). The variance of the surrogate was subtracted from that of the actual correlation pattern to control for the finite number of events recorded during imaging. **f.** Variance of correlations as a function of distance for all cortical areas after subtracting surrogate values. Data shown as mean ± SEM across all FOVs within an area. **g.** Correlation variance 2 mm from seed points is similar across cortical areas (KW: H(4) = 5.34 p = 0.254). Filled circles (24 of 25 FOVs) indicate experiments with statistically significant correlations at 2 mm relative to surrogate controls (dashed line at zero).

Figure S6

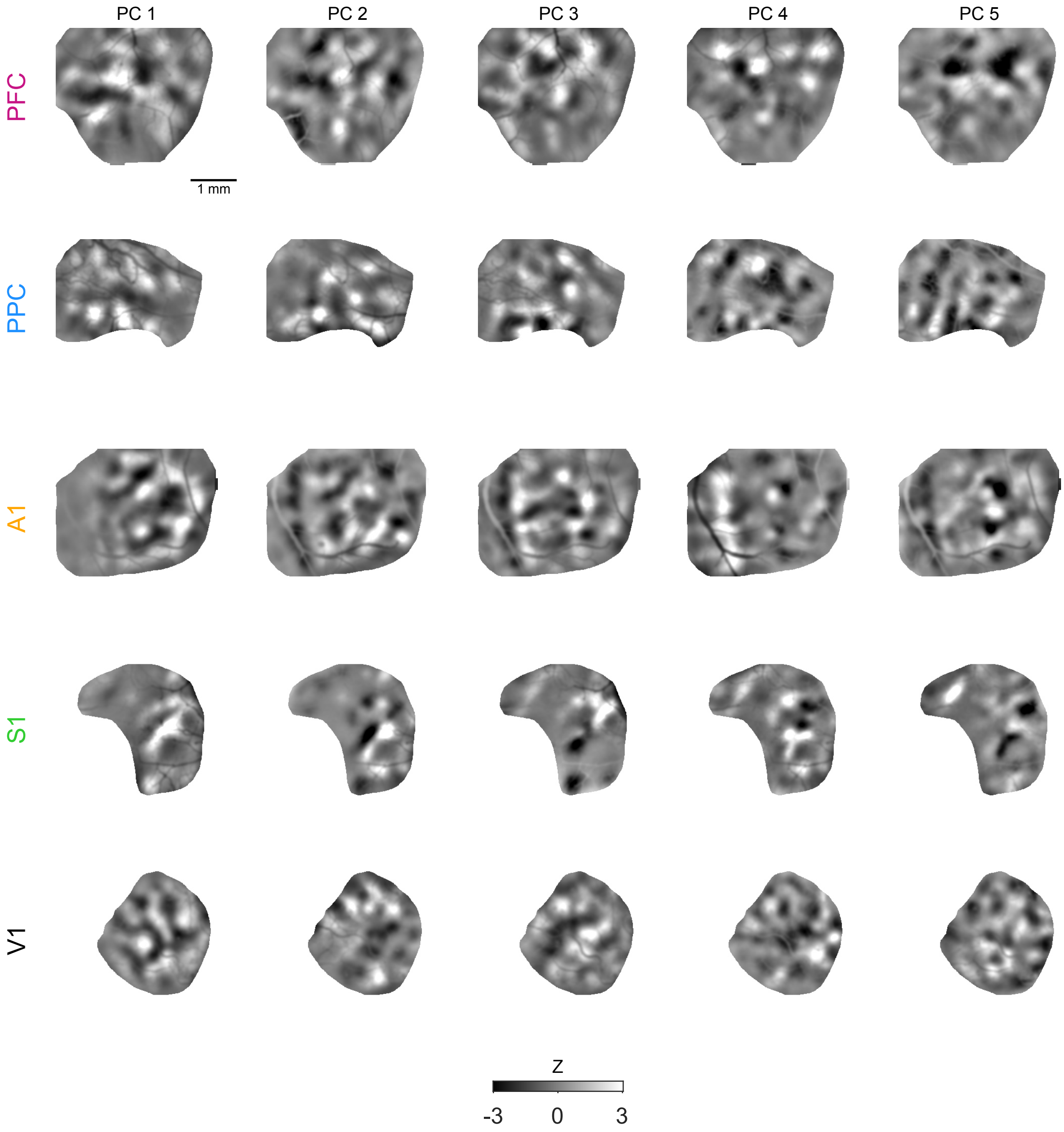

Fig. S6. Principal components of spontaneous activity are modular. Figure shows the first 5 principal components of spontaneous activity for the example experiments shown in Figure 2.

Figure S7

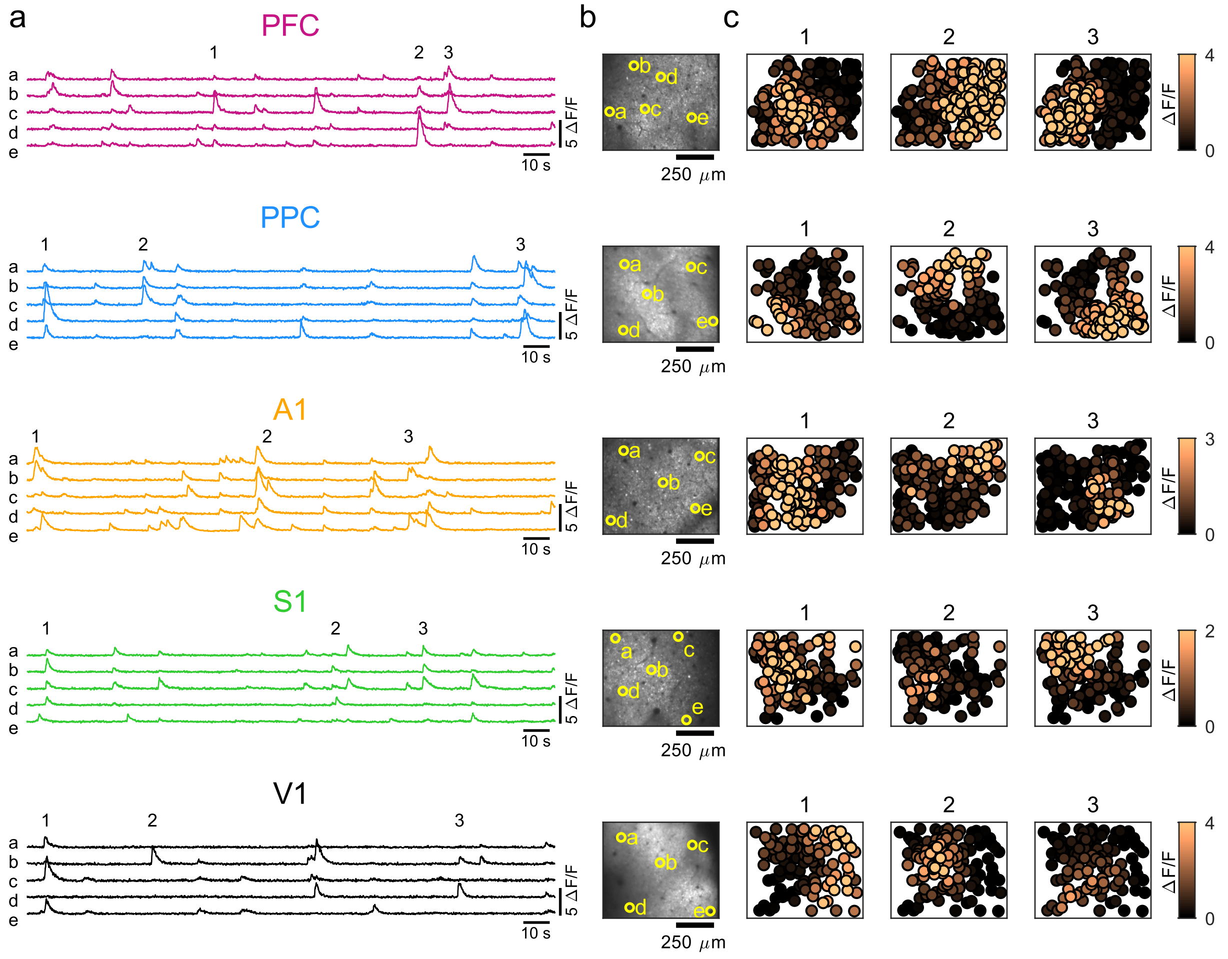

Fig. S7. Modular spontaneous events at cellular level across brain areas. For all brain areas imaged: **a.** Time course of cellular spontaneous activity extracted from 2-photon recordings. Numbers indicate events shown in (c), letters indicate cells labeled in (b). **b.** Field of view showing location of 5 example cells shown in (a). **c.** Three spontaneous events at times indicated in (a).

Figure S8

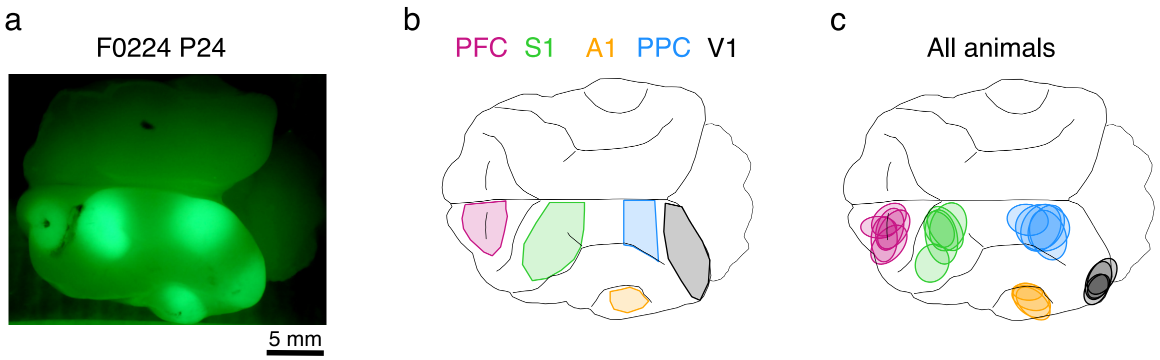

d

List of animals used in experiments

Animal	Areas	Injection Age	Imaging Age
F0155	PFC,PPC	13	23
F0223	A1,PFC,PPC,S1,V1	14	22
F0224	A1,PFC,PPC,S1,V1	15	24
F0231	A1,PPC,S1	14	22
F0232	PFC,S1,V1	14	21
F0233	PFC,S1,V1	14	23
F0234	A1,PFC,PPC	14	24
F0263	V1	14	21

Fig. S8. Histological reconstruction of imaged locations. a. Whole brain image from P24 animal with GCaMP expressing in PFC, S1, A1, PPC, and V1. In most experiments, only a subset of cortical areas were labeled and imaged in a given animal (range: 1-5, median 3). **b.** Region locations represented on a model brain image. **c.** Imaged locations reconstructed from histology for all animals, colored based on assigned cortical area. **d.** List of all animals with areas imaged and ages for all experiments.

Table S1-2

Table 1: Number of widefield events by area

Area	N animals	Total events	Median events	Range
PFC	6	1252	175	119 - 422
PPC	5	1228	231	166 - 399
A1	4	1931	412	233 - 874
S1	5	1092	253	100 - 324
V1	5	1971	424	165 - 521

Table 2: Number of 2-photon events by area

Area	N animals	N FOVs	Total events	Median events	Range
PFC	6	10	589	58	15 - 111
PPC	4	5	423	77	47 - 150
A1	4	5	419	78	64 - 117
S1	4	7	645	75	44 - 181
V1	5	6	508	82.5	42 - 128

Table S1: Number of spontaneous events recorded with widefield imaging across areas. Table lists number of animals, total number of events across all animals, median number of events per animal, and range across animals.

Table S2: Number of spontaneous events recorded with 2-photon imaging across areas. Table lists numbers of animals and FOVs, total number of events across all animals, median number of events per animal, and range across animals.

Table S3-6

Table 3: Module amplitude (widefield): post-hoc Conover-Iman test (related to Figure 1g)

Area	PPC	A1	S1	V1
PFC	p = 0.1786	p = 0.0915	p = 0.4009	p = 0.0011 *
PPC		p = 0.5427	p = 0.0240 *	p = 0.1086
A1			p = 0.0101 *	p = 0.2985
S1				p = 0.0001 *

Table 4: Dimensionality (widefield): post-hoc Conover-Iman test (related to Figure 2c)

Area	PPC	A1	S1	V1
PFC	p = 0.2847	p = 0.7282	p = 1.0000	p = 0.1053
PPC		p = 0.6578	p = 0.0977	p = 1.0000
A1			p = 0.2793	p = 1.0000
S1				p = 0.0290 *

Table 5: Local correlation strength (2-p): post-hoc Conover-Iman test (related to Figure 3d)

Area	PPC	A1	S1	V1
PFC	p = 0.0984	p = 0.7697	p = 0.0114 *	p = 0.2760
PPC		p = 0.3030	p = 0.0001 *	p = 0.9831
A1			p = 0.0208 *	p = 0.5680
S1				p = 0.0003 *

Table 6: Local coherence index (2-p): post-hoc Conover-Iman test (related to Figure 3e)

Area	PPC	A1	S1	V1
PFC	p = 0.5332	p = 0.9273	p = 0.3719	p = 1.0000
PPC		p = 0.7049	p = 0.0168 *	p = 1.0000
A1			p = 0.4589	p = 1.0000
S1				p = 0.2041

Table S3-6: Tables showing p-values for all pairwise comparisons for measures with significant group differences (Kruskal Wallis, p<0.05). Post-hoc tests were performed as described in Methods, via the Conover-Iman test with Holm's correction for multiple comparisons.

SI Movies 1-5: Spontaneous activity imaged in PFC, PPC, A1, S1, and V1, respectively. Movies show 10s of activity, in real-time (1x speed). Movies are shown as $\Delta F/F$. Scale bar: 1 mm.

Movie S1: Spontaneous activity in PFC.

Movie S2: Spontaneous activity in PPC.

Movie S3: Spontaneous activity in A1.

Movie S4: Spontaneous activity in S1.

Movie S5: Spontaneous activity in V1.
